# Challenges in multi-centric generalization: phase and step recognition in Roux-en-Y gastric bypass surgery

**DOI:** 10.1007/s11548-024-03166-3

**Published:** 2024-05-18

**Authors:** Joël L. Lavanchy, Sanat Ramesh, Diego Dall’Alba, Cristians Gonzalez, Paolo Fiorini, Beat P. Müller-Stich, Philipp C. Nett, Jacques Marescaux, Didier Mutter, Nicolas Padoy

**Affiliations:** 1 University Digestive Health Care Center - Clarunis, 4002 Basel, Switzerland; 2 Department of Biomedical Engineering, University of Basel, 4123 Allschwil, Switzerland; 3 Institute of Image-Guided Surgery, IHU Strasbourg, 67000 Strasbourg, France; 4 ICube, University of Strasbourg, CNRS, 67000 Strasbourg, France; 5 Altair Robotics Lab, University of Verona, 37134 Verona, Italy; 6 University Hospital of Strasbourg, 67000 Strasbourg, France; 7 Department of Visceral Surgery and Medicine, Inselspital Bern University Hospital, 3010 Bern, Switzerland; 8 IRCAD France, 67000 Strasbourg, France

**Keywords:** Surgical data science, Multi-centric validation, Gastric bypass, Phase recognition, Step recognition, Multi-task temporal convolutional network

## Abstract

**Purpose:**

Most studies on surgical activity recognition utilizing artificial intelligence (AI) have focused mainly on recognizing one type of activity from small and mono-centric surgical video datasets. It remains speculative whether those models would generalize to other centers.

**Methods:**

In this work, we introduce a large multi-centric multi-activity dataset consisting of 140 surgical videos (MultiBypass140) of laparoscopic Roux-en-Y gastric bypass (LRYGB) surgeries performed at two medical centers, i.e., the University Hospital of Strasbourg, France (StrasBypass70) and Inselspital, Bern University Hospital, Switzerland (BernBypass70). The dataset has been fully annotated with phases and steps by two board-certified surgeons. Furthermore, we assess the generalizability and benchmark different deep learning models for the task of phase and step recognition in 7 experimental studies: (1) Training and evaluation on BernBypass70; (2) Training and evaluation on StrasBypass70; (3) Training and evaluation on the joint MultiBypass140 dataset; (4) Training on BernBypass70, evaluation on StrasBypass70; (5) Training on StrasBypass70, evaluation on BernBypass70; Training on MultiBypass140, (6) evaluation on BernBypass70 and (7) evaluation on StrasBypass70.

**Results:**

The model’s performance is markedly influenced by the training data. The worst results were obtained in experiments (4) and (5) confirming the limited generalization capabilities of models trained on mono-centric data. The use of multi-centric training data, experiments (6) and (7), improves the generalization capabilities of the models, bringing them beyond the level of independent mono-centric training and validation (experiments (1) and (2)).

**Conclusion:**

MultiBypass140 shows considerable variation in surgical technique and workflow of LRYGB procedures between centers. Therefore, generalization experiments demonstrate a remarkable difference in model performance. These results highlight the importance of multi-centric datasets for AI model generalization to account for variance in surgical technique and workflows. The dataset and code are publicly available at https://github.com/CAMMA-public/MultiBypass140.

**Supplementary Information:**

The online version contains supplementary material available at 10.1007/s11548-024-03166-3.

## Introduction

The emerging field of Surgical Data Science (SDS) aims to impact the quality of interventional healthcare by collecting, organizing, analyzing, and modeling surgical data [1]. A principal element of SDS is to model surgical workflows which eventually could improve patient outcomes by providing intraoperative assistance, streamlining surgical training [2], preoperative planning, and postoperative analysis.

SDS has proposed systematic decomposition of workflows’ multi-level activities—whole procedure, phases, stages, steps, and actions [3]—and developed various methods to recognize these activities from endoscopic videos [4]. Recognition of phases [4–6], steps [6, 7], action triplets [8], and detection and localization of surgical tools [9, 10] are some of the popular tasks studied in the community.

**Fig. 2:**
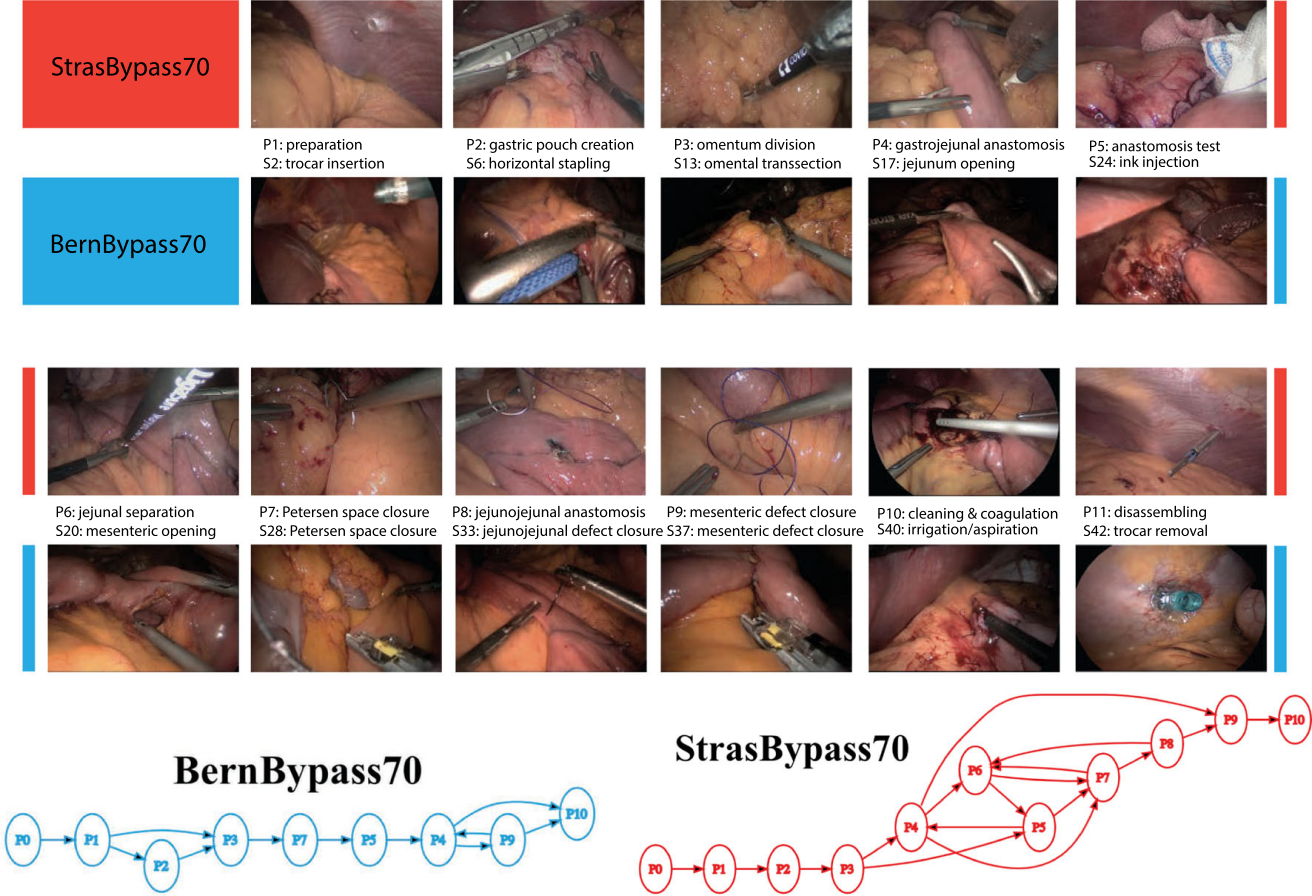
MultiBypass140: Sample video frames from StrasBypass70 and BernBypass70. (Bottom) Surgical workflow (modeled as phases [11]) followed in more than 10 surgeries in each medical center

Given the data-driven nature of these recent AI methods, the availability of large labeled surgical video datasets is paramount. Datasets have been curated to study phase recognition across different types of surgeries: Cholec80 [5] for laparoscopic cholecystectomy (LC), Bypass40 [6] for laparoscopic Roux-en-Y gastric bypass (LRYGB), laparoscopic sleeve gastrectomy [12], transanal total mesorectal excision [13], and laparoscopic inguinal hernia repair [14]. Nevertheless, datasets to train AI models for more fine-grained tasks, such as recognition of steps, action triplets, and safe dissection zones, have only been collected for specific surgeries. For example, Bypass40 [6] and CATARACTS^1^ have been annotated with steps for LRYGB and cataract surgeries, CholecT50 [8] contains surgical action triplets labels for LC and safe dissection zones have been studies for LC [15]. Furthermore, these labeled datasets have been collected from a single medical center. Training on mono-centric datasets limits the model’s generalizability to datasets from other centers. To overcome this generalization gap, multi-centric datasets representing different surgical techniques and workflows are warranted [16–18]. However, multi-centric datasets are rare as they are difficult to acquire and annotate consistently.

Besides, only a few works have explored recognizing activities at different levels of granularity. [6, 19] have attempted joint phase and step recognition using endoscopic video datasets from a single medical center. The most closely related work to this paper in objectives is HeiChole [18] which created a multi-centric dataset of 33 videos for phase recognition, action recognition, instrument detection, and skill assessment tasks. To date and to the best of our knowledge, phase and step recognition have not been studied in a multi-centric dataset of endoscopic videos.

To this end, the study has two objectives: creating a large multi-centric dataset for a complex LRYGB surgical procedure and recognizing activities at multiple levels. Thus, the contributions of this work are threefold:

1. Introduction of a multi-centric dataset of 140 LRYGB videos from two centers (Strasbourg and Bern).
2. The full annotated dataset with LRYGB ontology of 12 phases and 46 steps.
3. Evaluation of AI models for phase and step recognition and assessment of multi-centric model generalization.

## Datasets and annotations

**BernBypass70** dataset consists of 70 surgical videos of LRYGB at Inselspital, Bern University Hospital, Switzerland. The surgeries were performed by three surgeons. The videos were recorded at a resolution of $720\times 576$ at 25 frames-per-second (fps).

**StrasBypass70**, extending the Bypass40 [6] dataset, is a collection of 70 videos of LRYGB surgeries performed by surgeons at the University Hospital of Strasbourg, France. The videos were recorded at a resolution of $854\times 480$ or $1920\times 1080$ at 25 fps and were uniformly edited to $854\times 480$.

**MultiBypass140** is the combined dataset of 140 videos from Bern and Strasbourg university hospitals. Sample images of the two datasets are presented in Fig. 1. All videos have been anonymized by blacking out the out-of-body frames. Those out-of-body frames were detected using OoBNet [20] and verified by manual review.

**Annotations.** Two board-certified surgeons with more than 10 years of clinical practice annotated the MultiBypass140 dataset with activities at two levels of granularity, i.e., phases and steps. The annotation ontology of the LRYGB procedure as defined in [11] consists of 12 phases and 46 finer-grained steps. A detailed description of all the phases and steps can be found in the supplementary. MultiBypass140 was annotated using the MOSaiC software [21].

**Data Statistics.** On average, the surgical duration is 110 and 72 min and the total number of frames at 1 fps amounts to 464,794 and 305,907 in the StrasBypass70 and BernBypass70, respectively. Data characteristics of the multi-center dataset can be found in the supplementary. According to video duration, StrasBypass70 and BernBypass70 were split into training (40 videos), validation (10 videos), and test set (20 videos), resulting in 80 training, 20 validation, and 40 test videos for MultiBypass140.

### Model architecture

**Fig. 3:**
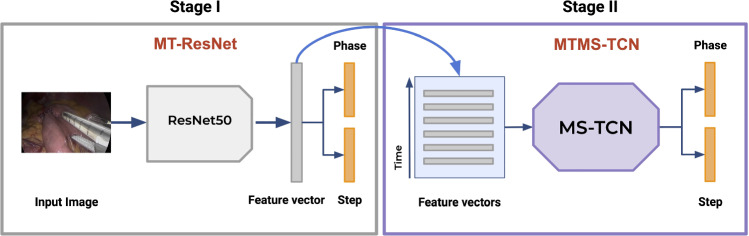
Schematic representation of MTMS-TCN. Stage I: the input images are processed by a ResNet-50 to extract visual features. Stage II: features of subsequent images of a video are stacked and processed by an MS-TCN for temporal awareness

MTMS-TCN [6], a state-of-the-art AI model for surgical activity recognition, was used for the experiments presented in this paper. The pipeline of MTMS-TCN consists of two stages where first a multi-task Convolutional Neural Network (CNN) (ResNet-50 [22]) model is employed for extracting visual features from images followed by a multi-task multi-stage Temporal Convolutional Network (TCN) to refine the features and extracting temporal information for joint phase and step recognition, as shown in Fig. 2.

**Fig. 4:**
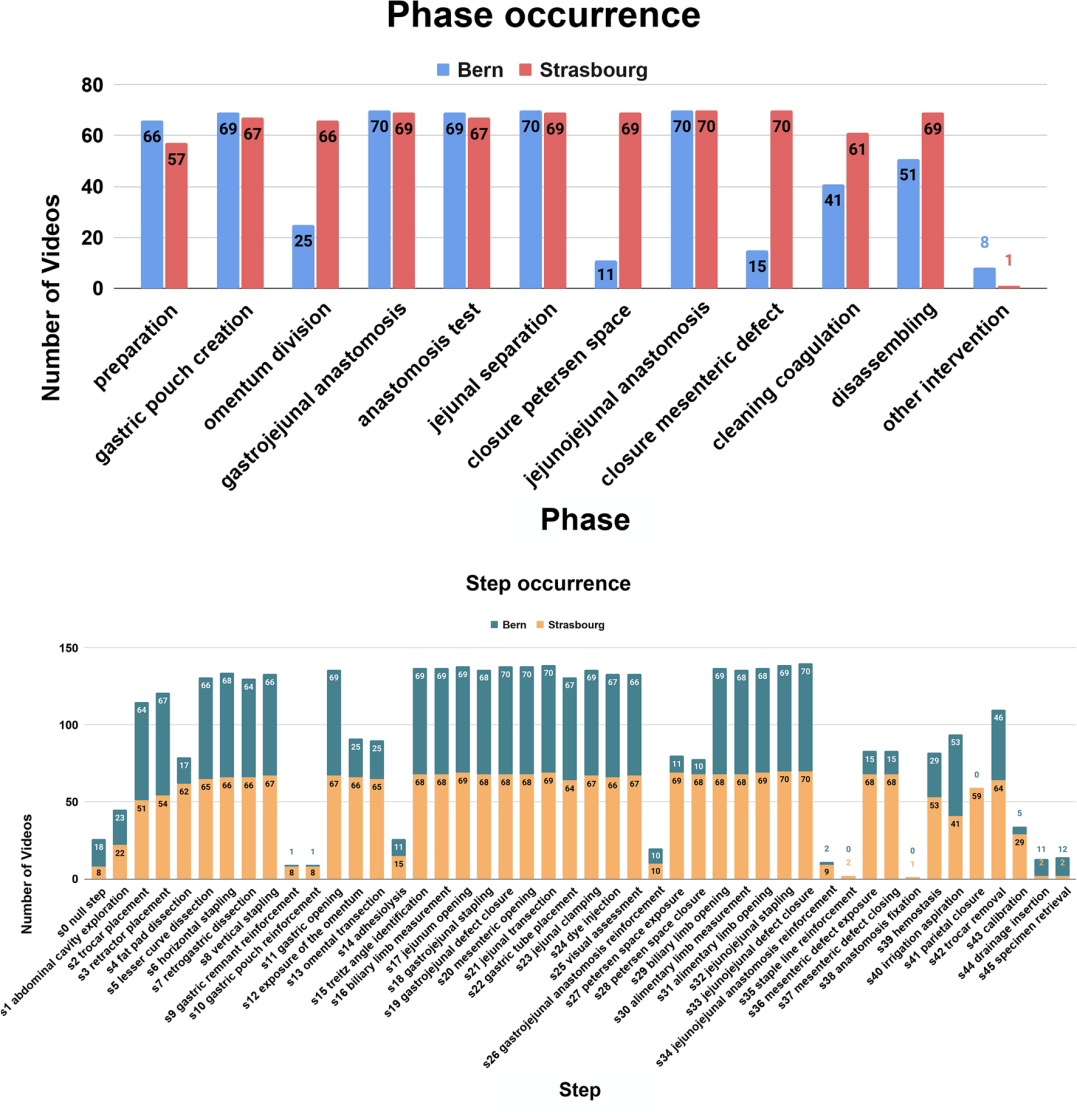
Total occurrence of phases and steps in the videos from the two medical centers

**Spatial model:** ResNet-50, a popular CNN architecture heavily employed for activity recognition, is utilized as a visual feature extractor and trained in multi-task learning of phase and step recognition. The model was initialized with pre-trained ImageNet weights and trained using Adam optimizer for 30 epochs.

**Temporal model:** MTMS-TCN [6] is a two-stage TCN model trained for 200 epochs in a multi-task learning setup on video features extracted from the CNN model. Furthermore, each stage of the TCN model consists of causal convolutions that utilize only information from past frames and dilated convolutions with exponentially increasing dilation factor for capturing long temporal dependencies.

**Table 1:** Benchmark of phase and step recognition. (Best results are in bold)

Phase					
Dataset	Model	ACC (%)	PR (%)	RE (%)	F1 (%)
	CNN	$74.53 \pm 13.34$	$44.79 \pm 8.44$	$45.69 \pm 8.19$	$42.38 \pm 9.14$
	LSTM	$79.73 \pm 13.75$	$54.91 \pm 9.31$	$56.19 \pm 10.24$	$52.60 \pm 10.34$
(1) BernBypass70	MT-LSTM	$80.69 \pm 13.85$	$56.98 \pm 11.54$	$57.14 \pm 13.38$	$54.15 \pm 12.84$
	TeCNO	$83.81 \pm 13.55$	$61.28 \pm 13.84$	$62.81 \pm 14.07$	$59.22 \pm 14.56$
	MTMS-TCN	${\textbf {85.30}} \pm {\textbf {13.19}}$	${\textbf {64.62}} \pm {\textbf {11.33}}$	${\textbf {67.41}} \pm {\textbf {13.81}}$	${\textbf {62.40}} \pm {\textbf {12.87}}$
	CNN	$82.46 \pm 7.90$	$72.91 \pm 9.17$	$73.37 \pm 8.67$	$71.13 \pm 9.47$
	LSTM	$86.37 \pm 7.68$	$76.66 \pm 9.52$	$80.90 \pm 9.63$	$76.42 \pm 10.35$
(2) StrasBypass70	MT-LSTM	$86.16 \pm 8.61$	$79.87 \pm 9.31$	$79.16 \pm 8.94$	$77.45 \pm 10.06$
	TeCNO	$89.50 \pm 7.55$	${\textbf {81.17}} \pm {\textbf {8.54}}$	${\textbf {84.26}} \pm {\textbf {7.73}}$	${\textbf {80.70}} \pm {\textbf {8.81}}$
	MTMS-TCN	${\textbf {90.23}} \pm {\textbf {7.04}}$	$80.48 \pm 9.37$	$82.39 \pm 8.22$	$79.87 \pm 9.37$
	CNN	$78.18 \pm 11.21$	$57.43 \pm 15.87$	$56.85 \pm 15.36$	$54.8 \pm 15.63$
	LSTM	$82.56 \pm 11.89$	$68.18 \pm 14.11$	$68.15 \pm 13.8$	$65.02 \pm 14.22$
(3) MultiBypass140	MT-LSTM	$83.94 \pm 11.18$	$67.58 \pm 14.93$	$66.88 \pm 15.67$	$64.86 \pm 15.97$
	TeCNO	$86.44 \pm 10.77$	${\textbf {72.59}} \pm {\textbf {13.99}}$	${\textbf {75.3}} \pm {\textbf {12.35}}$	$71.03 \pm 14.02$
	MTMS-TCN	${\textbf {87.91}} \pm {\textbf {10.64}}$	$72.27 \pm 13.13$	$74.82 \pm 13.36$	${\textbf {71.28}} \pm {\textbf {13.96}}$

Step					
Dataset	Model	ACC (%)	PR (%)	RE (%)	F1 (%)
	CNN	$58.92 \pm 11.63$	$38.26 \pm 7.95$	$38.47 \pm 7.39$	$35.55 \pm 7.44$
	LSTM	$64.99 \pm 12.44$	$48.66 \pm 10.91$	$48.66 \pm 11.12$	$44.88 \pm 10.53$
(1) BernBypass70	MT-LSTM	$63.54 \pm 13.92$	$49.40 \pm 11.21$	$48.49 \pm 12.37$	$44.93 \pm 11.48$
	TeCNO	$67.54 \pm 13.49$	$50.47 \pm 10.42$	${\textbf {53.01}} \pm {\textbf {11.74}}$	$47.56 \pm 10.85$
	MTMS-TCN	${\textbf {67.54}} \pm {\textbf {13.28}}$	${\textbf {51.04}} \pm {\textbf {10.36}}$	$52.84 \pm 10.44$	${\textbf {47.99}} \pm {\textbf {10.23}}$
	CNN	$70.44 \pm 11.48$	$50.29 \pm 7.1$	$50.66 \pm 8.4$	$47.67 \pm 8.19$
	LSTM	$75.26 \pm 11.67$	$60.15 \pm 7.35$	$58.74 \pm 9.04$	$56.37 \pm 9.05$
(2) StrasBypass70	MT-LSTM	$74.67 \pm 11.48$	$58.98 \pm 8.10$	$59.27 \pm 9.73$	$56.10 \pm 9.33$
	TeCNO	${\textbf {78.49}} \pm {\textbf {9.43}}$	${\textbf {60.15}} \pm {\textbf {6.92}}$	${\textbf {62.09}} \pm {\textbf {8.11}}$	${\textbf {58.13}} \pm {\textbf {7.87}}$
	MTMS-TCN	$77.78 \pm 10.24$	$59.14 \pm 7.84$	$61.28 \pm 8.65$	$57.27 \pm 8.47$
	CNN	$65.21 \pm 12.75$	$44.19 \pm 10.07$	$44.47 \pm 10.55$	$41.47 \pm 10.31$
	LSTM	$70.18 \pm 13.04$	$54.74 \pm 11.71$	$54.24 \pm 12.55$	$51.15 \pm 12.35$
(3) MultiBypass140	MT-LSTM	$69.55 \pm 13.76$	$53.92 \pm 11.64$	$53.14 \pm 12.64$	$50.11 \pm 12.45$
	TeCNO	${\textbf {73.49}} \pm {\textbf {13.17}}$	${\textbf {55.81}} \pm {\textbf {11.1}}$	${\textbf {57.29}} \pm {\textbf {12.18}}$	${\textbf {53.08}} \pm {\textbf {11.95}}$
	MTMS-TCN	$72.85 \pm 12.68$	$55.32 \pm 10.55$	$56.58 \pm 11.7$	$52.59 \pm 11.32$

### Experiments

To benchmark phase and step recognition on BernBypass70, StrasBypass70, and on the joint MultiBypass140 dataset, five different model architectures were assessed: (1) ResNet-50 (CNN) [22], (2) long short-term memory (LSTM) [23], (3) Multi-task LSTM (MT-LSTM), (4) multi-stage TCN (TeCNO) [24], and (5) MTMS-TCN  [6].

Seven experimental setups were used to analyze the generalizability of AI models:

1. Training and evaluation on BernBypass70
2. Training and evaluation on StrasBypass70
3. Training and evaluation on the joint MultiBypass140
4. Training on BernBypass70 and evaluation on StrasBypass70
5. Training on StrasBypass70 and evaluation on BernBypass70
6. Training on MultiBypass140 and evaluation on BernBypass70
7. Training on MultiBypass140 and evaluation on StrasBypass70

### Model evaluation

Model performance was assessed by comparing human ground truth annotations with model predictions measuring accuracy, precision, recall, and F1-score. Following previous works, performance metrics were averaged across phases and steps per video and then across videos [6, 24].

**Fig. 5:**
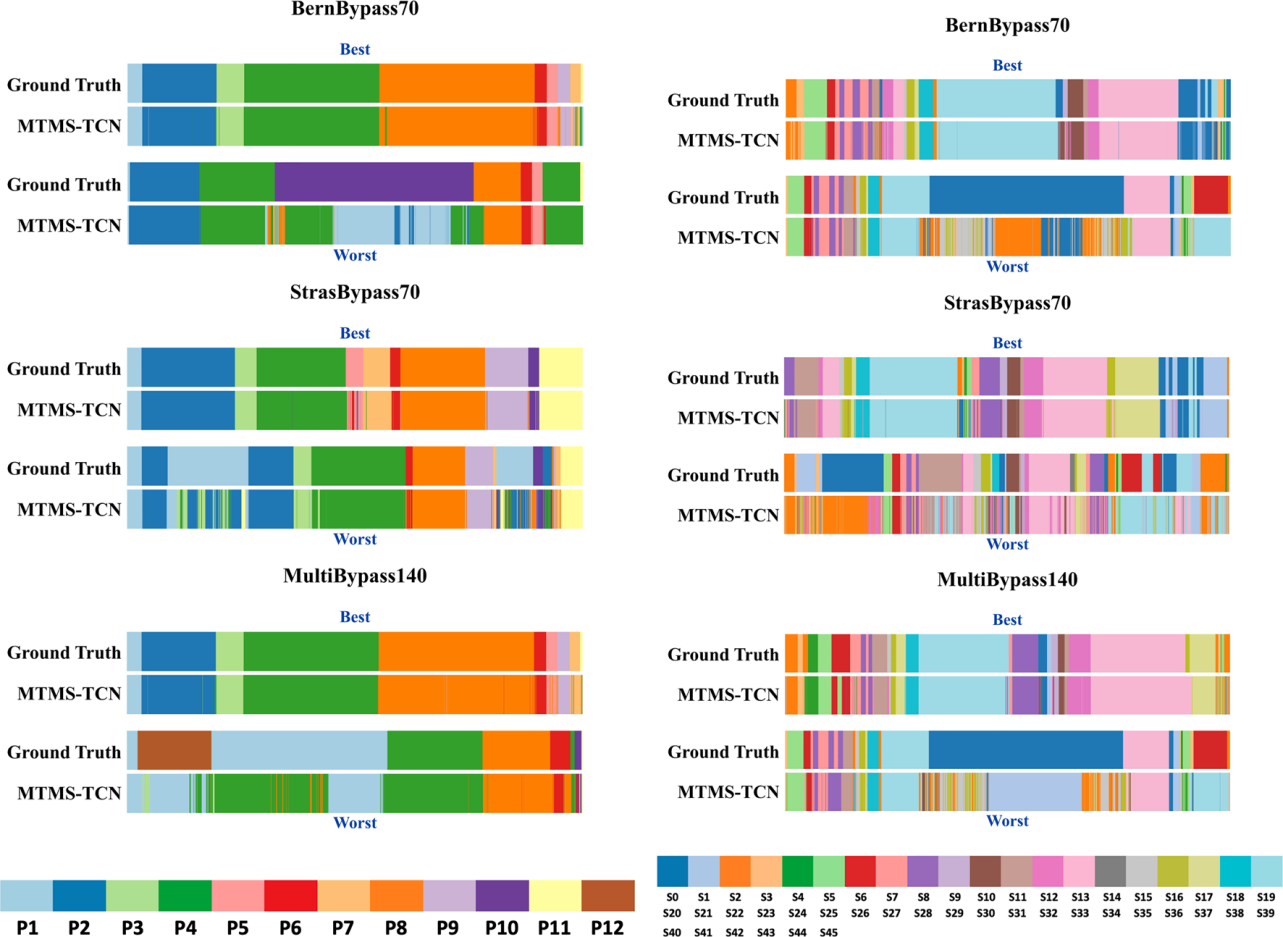
Best (upper row) and worst (lower row) video pairs of ground truth annotations (top) and MTMS-TCN predictions (bottom) for all 3 datasets. The width of each phase is relative to its duration

## Results & discussions

This is the first study to evaluate AI models for multi-level activity recognition, i.e., phases and steps, on a large multi-centric video dataset of LRYGB procedures. In this section, we present the results and discuss our findings.

**Workflow: Strasbourg vs Bern.** Differences in surgical workflow between medical centers are common, as different surgeons perform the interventions. StrasBypass70 has an average video duration of 111±33 min consisting of 10 phases and 33 steps. BernBypass70 has an average video duration of 73±20 min consisting of 8 phases and 27 steps. To understand the LRYGB surgical workflow differences between centers, we visualize the phase and step occurrences in Fig. 3 and the surgical workflows, modeled as phase transition graphs, in Fig. 1.

In StrasBypass70, the occurrence of phases and steps is evenly distributed. Either a phase or a step occurs in most videos, or it does not occur at all. In contrast, BernBypass70 has only some videos containing all phases and steps. Most of the videos contain a subset of phases and steps. These differences in dataset distribution of phases and steps between centers result from differences in surgical technique and workflows. In StrasBypass70, the omentum is routinely divided (P3) and both mesenteric defects are routinely closed (P7 & P9), which is not routinely done in BernBypass70. Given the hierarchical structure of phases and steps, with every phase missing, corresponding steps are missing as well. Hence, the average video of BernBypass70 contains 2 phases and 6 steps less than the average StrasBypass70 video. This finding is also reflected by the average video duration which is 38 min shorter in BernBypass70 compared to StrasBypass70 videos.

**Recognition: Individual centers.** To independently analyze the performance of AI models on each center/dataset, we train different models on BernBypass70, StrasBypass70, and MultiBypass140 datasets and evaluate the models’ performance on respective test sets. The phase and step recognition task results are presented in Table 1.

**Table 2:** Cross dataset evaluation of MTMS-TCN

Experiment	Model	ACC (%)	PR (%)	RE (%)	F1 (%)
Phase					
(4) BernBypass70 $\rightarrow$	CNN	$57.34 \pm 8.52$	$35.94 \pm 6.16$	$45.41 \pm 6.51$	$32.72 \pm 5.47$
StrasBypass70	MTMS-TCN	$64.44 \pm 7.91$	$36.76 \pm 5.49$	$40.16 \pm 7.38$	$33.10 \pm 5.72$
(5) StrasBypass70 $\rightarrow$	CNN	$56.66 \pm 14.48$	$32.14 \pm 7.61$	$34.13 \pm 7.36$	$29.54 \pm 8.21$
BernBypass70	MTMS-TCN	$72.36 \pm 17.57$	$42.21 \pm 9.80$	$45.13 \pm 13.55$	$39.05 \pm 11.95$
(6) MultiBypass140 $\rightarrow$	CNN	$76.77 \pm 12.34$	$46.48 \pm 7.41$	$46.90 \pm 8.72$	$43.99 \pm 8.29$
BernBypass70	MTMS-TCN	$85.62 \pm 12.74$	$62.13 \pm 8.34$	$65.02 \pm 10.56$	$60.63 \pm 9.49$
(7) MultiBypass140 $\rightarrow$	CNN	$83.30 \pm 8.03$	$70.85 \pm 8.18$	$71.70 \pm 8.36$	$69.46 \pm 8.75$
StrasBypass70	MTMS-TCN	$90.19 \pm 7.31$	$82.41 \pm 8.33$	$84.63 \pm 7.31$	$81.93 \pm 8.54$
Step					
(4) BernBypass70 $\rightarrow$	CNN	$40.16 \pm 9.65$	$26.12 \pm 4.55$	$27.82 \pm 5.65$	$20.99 \pm 4.36$
StrasBypass70	MTMS-TCN	$44.87 \pm 10.42$	$29.05 \pm 5.96$	$29.16 \pm 5.59$	$23.81 \pm 5.63$
(5) StrasBypass70 $\rightarrow$	CNN	$37.45 \pm 11.48$	$18.51 \pm 4.74$	$21.41 \pm 3.78$	$17.35 \pm 4.56$
BernBypass70	MTMS-TCN	$49.00 \pm 15.14$	$24.98 \pm 6.52$	$29.01 \pm 7.74$	$23.23 \pm 6.56$
(6) MultiBypass140 $\rightarrow$	CNN	$57.19 \pm 12.07$	$36.18 \pm 7.29$	$36.09 \pm 7.53$	$33.25 \pm 7.42$
BernBypass70	MTMS-TCN	$67.74 \pm 13.05$	$50.06 \pm 10.99$	$51.06 \pm 12.34$	$46.82 \pm 11.35$
(7) MultiBypass140 $\rightarrow$	CNN	$70.23 \pm 11.36$	$50.33 \pm 6.87$	$50.49 \pm 7.54$	$47.45 \pm 7.73$
StrasBypass70	MTMS-TCN	$77.96 \pm 9.96$	$60.59 \pm 6.83$	$62.11 \pm 7.78$	$58.35 \pm 7.80$

All the models, both spatial and spatio-temporal, achieve considerably low performance across all the metrics on BernBypass70 in comparison to StrasBypass70. For instance, the CNN (ResNet-50) spatial model on phase recognition task shows 8% lower accuracy and a staggering 28% degradation in F1-score on BernBypass70 compared to StrasBypass70. Spatio-temporal model, MTMS-TCN, performs 5% lower in accuracy and 15-17% lower on all other metrics on BernBypass70 over StrasBypass70. Similarly for step recognition, CNN and MTMS-TCN on BernBypass70 achieve 12% and 8-10% lower than StrasBypass70 on all metrics. These differences are direct consequences of the differences in surgical workflow followed in the two centers and consistent with previous work on LC [17]. Given that many phases and steps (Fig. 3) are not carried out routinely in Bern, their occurrences/class distribution is notably skewed in BernBypass70 which makes recognition of phases and steps increasingly challenging for AI models on this dataset. This can be witnessed in Fig. 4 where the model performs best on videos following common workflow (P1$\rightarrow$P2$\rightarrow$P3$\rightarrow$...) in both the datasets while performing worse when there is unexpected flow of phases/steps performed during surgeries (P4$\rightarrow$P10$\rightarrow$P8 or P1$\rightarrow$P12$\rightarrow$P1$\rightarrow$P4).

Lastly, all the AI models on the combined MultiBypass140 dataset have a performance exceeding the performance on BernBypass70, but inferior to the performance on StrasBypass70.

**Recognition: Cross-center.** To examine models’ ability to transfer knowledge learnt from one center to the other, we train CNN and MTMS-TCN on one center and evaluate them on the other (experiments 4, 5, 6, & 7). The experimental results are tabulated in Table 2.

The performance of the CNN & MTMS-TCN in these experiments is considerably inferior to training and evaluation on individual mono-centric datasets (experiments 1 & 2). CNN & MTMS-TCN trained on BernBypass70 when evaluated on StrasBypass70 without any fine-tuning achieve 57% & 64% in accuracy and 32% & 33% in F1 score for phase and step recognition. This is due to the significant differences in the workflow followed in Bern with many phases and steps not routinely carried out. Inversely, CNN & MTMS-TCN achieves 56% & 72% in accuracy and 29% & 39% in F1 when trained on StrasBypass70 and evaluated on BernBypass70. Although in StrasBypass70 the occurrence of phases and steps are evenly distributed, the knowledge learned by these models on StrasBypass70 is still not transferable to BernBypass70. This odd performance could be for two reasons: 1) The variability in visual appearance between centers caused due to different instruments, lighting, or patients’ demographics; 2) alongside this, the temporal differences caused due to changes in the surgical workflow across surgeons and medical centers.

Both CNN & MTMS-TCN trained on MultiBypass140 when evaluated on the mono-centric datasets (experiments 6 & 7) achieve performance close to its performance when trained and evaluated on the individual dataset (experiments 1 & 2) for both the phase and step recognition tasks. This shows the capacity of AI models to learn all the variations existing in the data and domain without compromising performance.

**Challenges.** Despite its multi-centric design, this study is limited by the fact that datasets from only two centers are involved. The significant variability in surgical technique and image domain makes the transferability of AI models between centers a challenging task. More studies on adding video datasets from other clinical centers are imperative to capture the variability in surgical technique and dataset distributions. Future studies should focus on developing AI models to learn from a large corpus of unlabeled data from multiple centers. MultiBypass140 is a starting point for studying objective metrics to quantify the variability of surgical workflows. These metrics can exploit quality/similarity measures of endoscopic images combined with similarity metrics between transition graphs at different levels of granularity, i.e. phases and steps.

## Conclusion

This study demonstrates the need to exhibit the variation of surgical techniques and workflow to develop generalizable AI models. With extensive experimentation, it has been shown that dataset distribution and size due to different LRYGB workflows between centers have a major impact on model performance. This work highlights the importance of multi-centric datasets for the training and evaluation of AI models in surgical video analysis.

## Supplementary Information

Below is the link to the electronic supplementary material.

Supplementary file 1 (pdf 159 KB)

## Footnotes

1 https://cataracts2020.grand-challenge.org/.
