# Supplementary material for: Challenges in multi-centric generalization: phase and step recognition in Roux-en-Y gastric bypass surgery
Source: Int J Comput Assist Radiol Surg. 2024 May 18;19(11):2249–57. doi: 10.1007/s11548-024-03166-3 (PMC11541311; doi:10.1007/s11548-024-03166-3)
Supplement: Supplementary file 1 — (pdf 159 KB) [file 11548_2024_3166_MOESM1_ESM.pdf]

Appendix

A. Phase and Step Definitions

All the 12 phases and their definitions have been tabulated in Table 1. Similarly, all the 46 steps of a laparoscopic Roux-en-Y gastric bypass, along with their definitions, are presented in Table 2.

Table 1: Definitions of the 12 phases of laparoscopic Roux-en-Y gastric bypass surgery.

Phase ID	Phase Name	Description
P1	Preparation	Access to the abdominal cavity, installation of the ports and exposure of the operating field
P2	Gastric pouch creation	The proximal part of the stomach is separated from the rest to create a gastric pouch
P3	Omentum division	Vertical transection of the omentum majus to facilitate the ascent of the small bowel to the gastric pouch
P4	Gastrojejunal anastomosis	Anastomosis of the small bowel with the gastric pouch
P5	Anastomosis test	Verification that the gastrojejunostomy does not leak
P6	Jejunal separation	Separation of the proximal alimentary limb and the biliary limb by transection of the jejunum
P7	Petersen space closure	Closure of the Petersen space between the alimentary limb and the transverse mesocolon
P8	Jejunojejunal anastomosis	Anastomosis of the distal alimentary limb with the biliary limb
P9	Mesenteric defect closure	Closure of the mesenteric defect at the jejunojejunostomy
P10	Cleaning & coagulation	Irrigation and aspiration of liquid/blood in the abdominal cavity, hemostasis
P11	Disassembling	Removal of the surgical instruments, retractor, ports, and camera
P12	Other intervention	If any additional intervention is performed (e.g. liver biopsy, cholecystectomy)

Table 2: Definitions of the 46 steps of laparoscopic Roux-en-Y gastric bypass surgery.

Step ID	Step Name	Description
S0	Null step	The camera is static and no actions are performed by the surgeon
S1	Abdominal cavity exploration	The abdominal cavity is explored to detect alterations that could modify or prevent the planned surgery
S2	Trocar placement	Accessory trocars are introduced into the abdominal cavity

Continued on next page

Table 2 – *Continued from previous page*

Step ID	Step Name	Description
S3	Retractor placement	Introduction and placement of a liver retractor to expose the esophagogastric junction
S4	Fat pad dissection	Dissection of the fatty tissue surrounding the esophagogastric junction to expose the angle of his and remove adhesions to the spleen
S5	Lesser curvature dissection	Opening of a retrogastric window at the lesser curvature of the stomach to facilitate the passage of the stapler
S6	Horizontal stapling	Horizontal transection of the stomach with the stapler starting from the lesser curvature to create the horizontal part of the pouch
S7	Retrogastric dissection	Dissection of the tissue dorsal to the stomach for better exposure
S8	Vertical stapling	Vertical transection of the stomach with the stapler to create the vertical portion of the pouch
S9	Gastric remnant reinforcement	Reinforcement of the gastric remnant staple line with a suture
S10	Gastric pouch reinforcement	Reinforcement of the gastric pouch staple line with a suture
S11	Gastric opening	Creation of an orifice into the gastric pouch where the gastrojejunostomy will be created
S12	Exposure of the omentum	Grasping and lifting of the omentum to expose it
S13	Omental transection	Transection of the omentum to divide it into two parts
S14	Adhesiolysis	Transection of connective tissue or adhesions
S15	Treitz angle identification	Visualization of the Treitz angle to identify the proximal jejunum
S16	Biliary limb measurement	Measurement of the small bowel length from Treitz angle to the future site of the gastrojejunostomy to determine the length of the biliary limb
S17	Jejunum opening	Opening of the distal jejunum where the gastrojejunostomy will be created
S18	Gastrojejunal stapling	Creation of the gastrojejunostomy using a stapler
S19	Gastrojejunal defect closure	Closure of the orifice left by the stapler creating the gastrojejunostomy
S20	Mesenteric opening	Opening of the mesentery to facilitate the introduction of the stapler
S21	Jejunal transection	Transection of the jejunum proximal to the gastrojejunostomy
S22	Gastric tube placement	Movement of the gastric tube (e.g. to calibrate the size of the gastric pouch or the gastrojejunostomy)
S23	Jejunal clamping	Clamping of the jejunum distal to the gastrojejunostomy

Continued on next page

Table 2 – *Continued from previous page*

Step ID	Step Name	Description
S24	Dye injection	Injection of dye (methylene blue) to detect any leakage of the gastrojejunostomy
S25	Visual assessment	Visual inspection of the anastomosis for any leakages
S26	Gastrojejunal anastomosis reinforcement	Reinforcement of the gastrojejunostomy with an additional suture
S27	Petersen space exposure	Exposure of the Petersen space (between the alimentary limb and the transverse colon
S28	Petersen space closure	Closing of the Petersen space with suture or staples
S29	Biliary limb opening	Opening of the biliary limb where the jejunojejunostomy will be created
S30	Alimentary limb measurement	Measurement of the small bowel length from the gastrojejunostomy to the future site of the jejunojejunostomy to determine the length of the alimentary limb
S31	Alimentary limb opening	Opening of the alimentary limb where the jejunojejunostomy will be created
S32	Jejunojejunal stapling	Creation of the jejunojejunostomy using a stapler
S33	Jejunojejunal defect closure	Closure of the orifice left by the stapler creating the jejunojejunostomy
S34	Jejunojejunal anastomosis reinforcement	Reinforcement of the jejunojejunostomy with an additional suture
S35	Staple line reinforcement	Staple line reinforcement of the blind limb of the jejunojejunostomy with an additional suture
S36	Mesenteric defect exposure	Exposure of the mesenteric defect created by the jejunojejunostomy
S37	Mesenteric defect closure	Closing of the mesenteric defect with suture or staples
S38	Anastomosis fixation	One or more stitches to fix the position of an anastomosis
S39	Hemostasis	Any intervention to stop bleeding
S40	Irrigation/Aspiration	Irrigation and aspiration of any liquid or blood clots to be removed from the abdominal cavity
S41	Parietal closure	Closure of the abdominal wall at the trocar sites
S42	Trocar removal	Removal of all the trocars and the liver retractor
S43	Suture of small bowel lesion	Rectification of a small bowel lesion using a suture
S44	Drainage insertion	Insertion of drainage into the abdominal cavity to drain fluids
S45	Specimen retrieval	Removal of any spare tissue (e.g. omentum, small bowel, or stomach)

B. Phase and Step Hierarchy

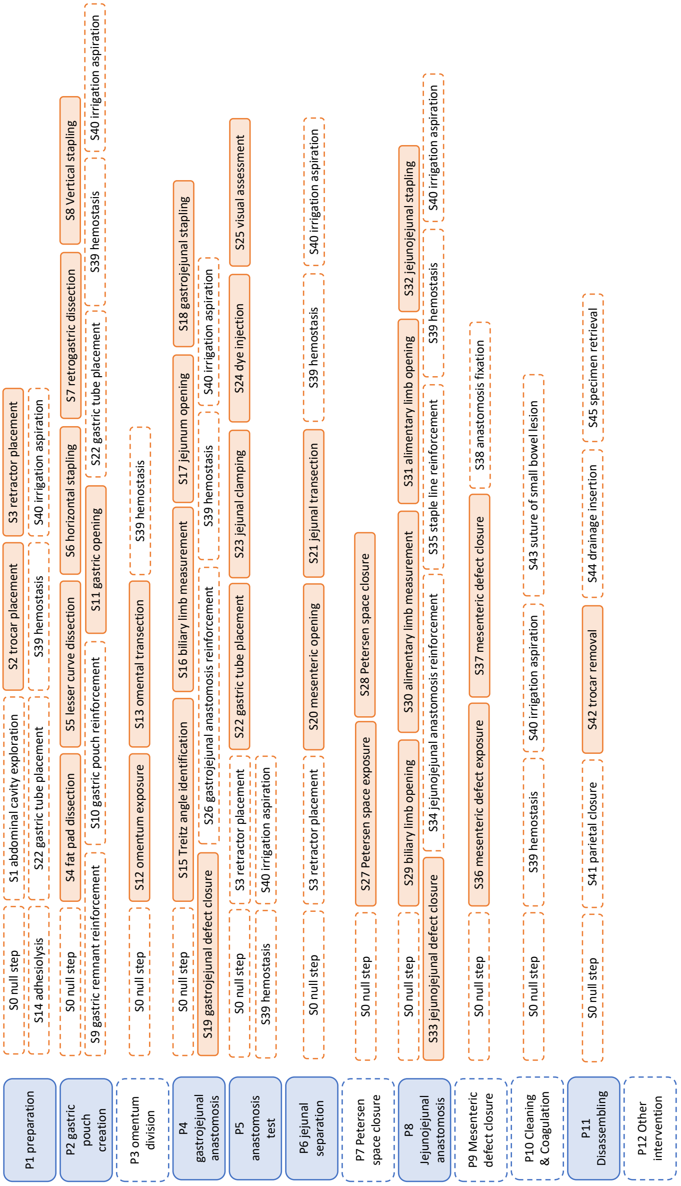

Fig. 1: Hierarchical structure of phases and steps in the laparoscopic Roux-en-Y gastric bypass ontology. Facultative phases and steps have a dashed border as published in [11].

C. Dataset Characteristics

Table 3: Data characteristics of the multi-center dataset presented in this work.

Dataset	Videos (n)	Min. duration (minutes)	Max. duration (minutes)	Mean ± std duration (minutes)	Total frames (n)
StrasBypass70					
Training	40	41	171	106 ± 32	253,913
Validation	10	78	176	121 ± 33	72,555
Test	20	63	178	115 ± 32	138,326
BernBypass70					
Training	40	37	114	69 ± 19	166,431
Validation	10	54	116	77 ± 20	46,497
Test	20	52	145	77 ± 24	92,979
MultiBypass140					
Training	80	37	171	88 ± 33	420,344
Validation	20	54	176	99 ± 35	119,052
Test	40	52	178	96 ± 34	231,305
